# Recurrent Unicystic Ameloblastoma of the Infratemporal and Temporal Fossa

**DOI:** 10.5005/jp-journals-10005-1039

**Published:** 2009-04-26

**Authors:** Sunil Sharma, Dinesh Kumar, Abhishek Vashistha, Urvashi Bihani, Mridula Trehan

**Affiliations:** 1 Vice Principal and Head, Department of Oral and Maxillofacial Surgery, Mahatma Gandhi Dental College, Jaipur, Rajasthan India; 2 Assistant Professor, Department of Oral and Maxillofacial Surgery, Mahatma Gandhi Dental College, Jaipur, Rajasthan, India; 3 Reader, Department of Oral and Maxillofacial Surgery, Mahatma Gandhi Dental College, Jaipur, Rajasthan, India; 4 Professor, Department of Oral and Maxillofacial Surgery, Mahatma Gandhi Dental College, Jaipur, Rajasthan, India; 5 Professor and Head, Department of Orthodontics, Mahatma Gandhi Dental College, Jaipur, Rajasthan, India

**Keywords:** Unicystic, ameloblastoma, infratemporal temporal.

## Abstract

The ameloblastoma has been described as benign but locally invasive; benign and locally invasive with strong tendency to recur; and locally malignant. Recurrence of this lesion in to the infratemporal and temporal region are rarely reported cases. Complete excision of lesion was done with the help of the advance imaging modalities and possible cause of recurrence in this case is discussed.

## INTRODUCTION

First introduced in 1977 by Robinson and Martinez and popularized by Gardner in 1983.

It has been used to describe an ameloblastoma developing within the lining, lumen, or wall of a cyst as well as an invasive ameloblastoma that has a single cystic space rather than multicystic spaces. In some publications the term has been used to describe an ameloblastoma limited to the cyst lining, an ameloblastoma solely within the cyst lumen and in still others, an ameloblastoma with varying degrees of invasion through the connective tissue layers of the cyst. No standard terminology to describe cystic ameloblastoma with limited invasion to aggressive invasion is followed, which as lead to inadequate curative surgical approaches and inaccurate recurrence rates.

## CASE REPORT

**Fig. 1.**
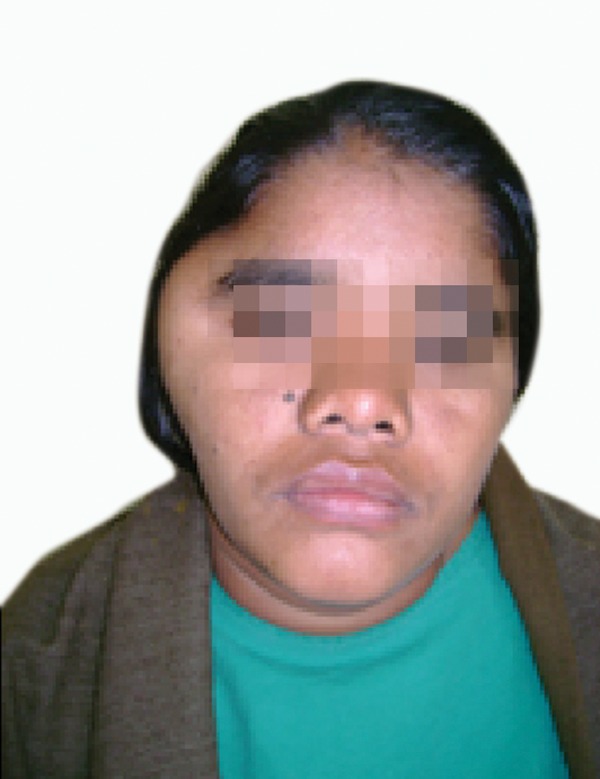
Preoperative picture showing right temporal swelling

**Fig. 2.**
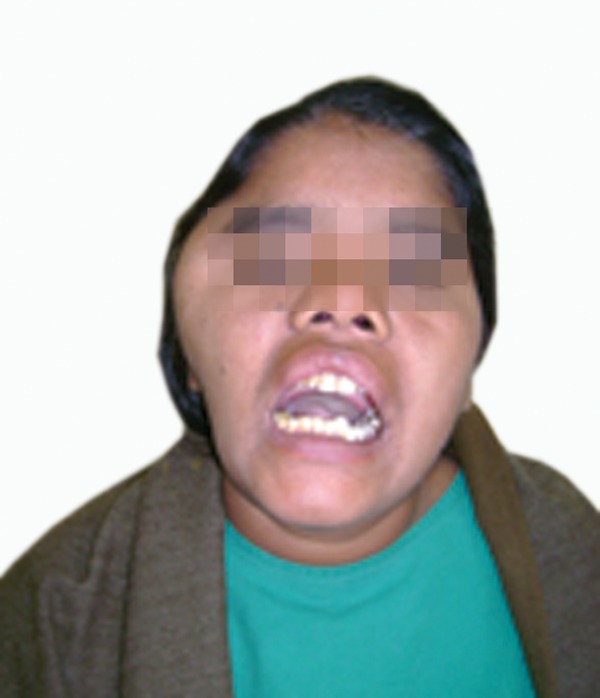
Decreased mouth opening

A 20 years old female patient reported to our maxillofacial unit with a swelling on the right temporal region of face since 18 months. The swelling increased gradually with no history of pus discharge or pain before and after the onset of swelling. A significant past history was reported from patient that she suffered a similar type of swelling on the right side of ramus of the mandible around 30 months back. Patient visited to a regional cancer center where a simple enucleation was done and reported histopathologically as radicular cyst. Six months following surgery patient started noticing a swelling in right temporal region which gradually increased to present size with gradual decrease in mouth opening (Figs 1 and 2).

### Clinical Features

- Swelling in the right temporal region extending anteriorly up to lateral orbital rim and zygomatic process of maxilla. Posteriorly up to root of the ear but not crossing it. Superiorly 5 cm above the root of the ear anteriorly up to temporal hair line and inferiorly 3 cm below the zygomatic arch.
- Size of the swelling around 10 × 5 cm (*l × b*)
- Uniform egg shaped swelling, smooth surface and skin over the swelling appear normal.
- No sinus opening, erythema or visible pulsation in the swelling.
- On palpation swelling is well-defined and skin over the swelling is movable, non-tender, bony hard swelling and fixed to the underlying tissues. No eggshell crackling or palpable pulsations elicited.
- Zygomatic arch appears to be expanded along with the swelling and right TMJ movement is not palpable.
- Angle and ramus of the mandible on affected side are not well-defined.
- No paresthesia of lip, check and temporal region on the affected side.
- Ears, eyes, nose—No abnormality detected on the affected side
- Lymph nodes are palpable, non-tender, not enlarged and mobile on affected side.
- Mouth opening 25 mm, poor oral hygiene with missing lower right second and third molars.
- Anterior border and lateral surface of the ramus is not palpable.
- Intraorally a bony hard swelling is palpable in the right coronoid region indicating its extension in to the right infratemporal space. No sinus or erythema in the mucosa over the swelling intraorally.
- Occlusion of teeth not deranged and no mobility of upper and lower teeth.
- Tongue, floor of the mouth, hard palate and soft palate no abnormality detected.

**Fig. 3.**
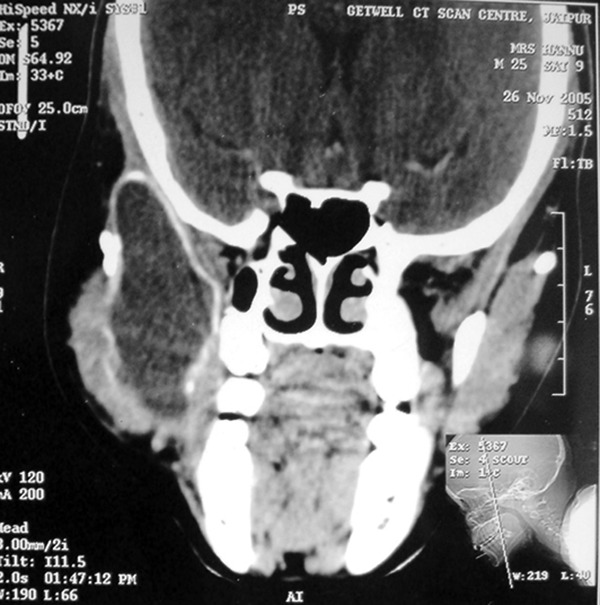
CT scan of lesion

### Radiographic Findings

CT scan of previously operated lesion at regional cancer center shows large expanded cystic lesion in the right ramus of the mandible extending from angle of the mandible inferiorly and involving the coronoid completely superiorly. Anteroposteriorly completely involving the ramus of the mandible (Fig. 3). Uniform thick cystic lining can be appreciated with thinned out bone margins.

**Fig. 4.**
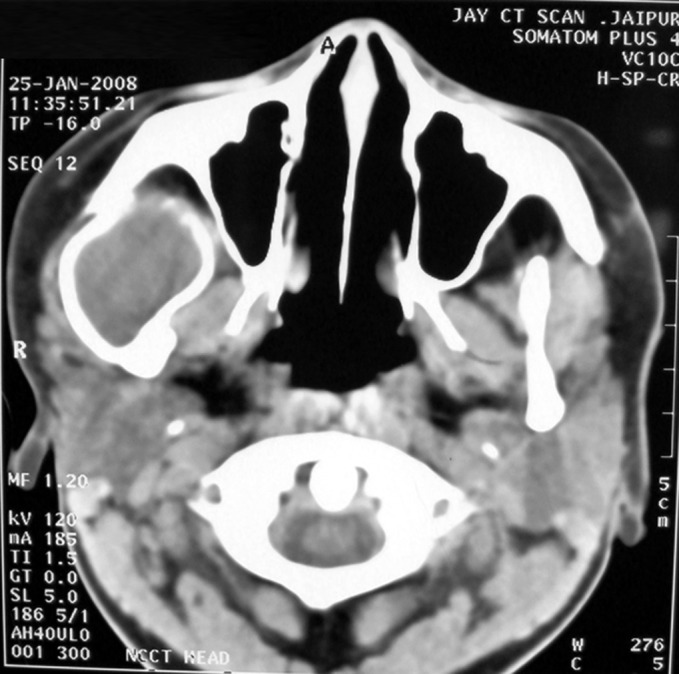
CT scan of secondary lesion showing involvement of condylar head

**Fig. 5.**
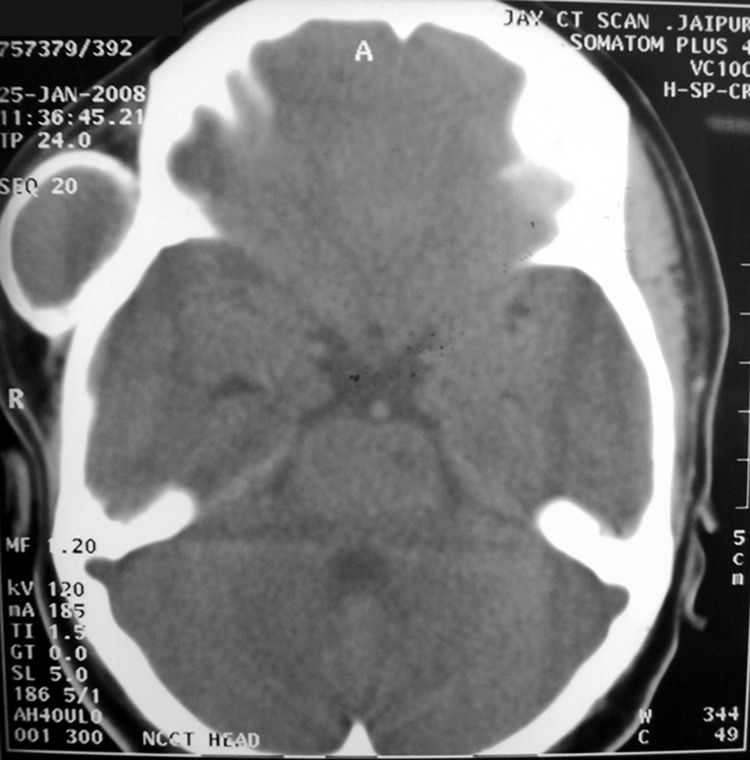
CT scan of secondary lesion in right temporal region showing bony cystic lesion

**Fig. 6.**
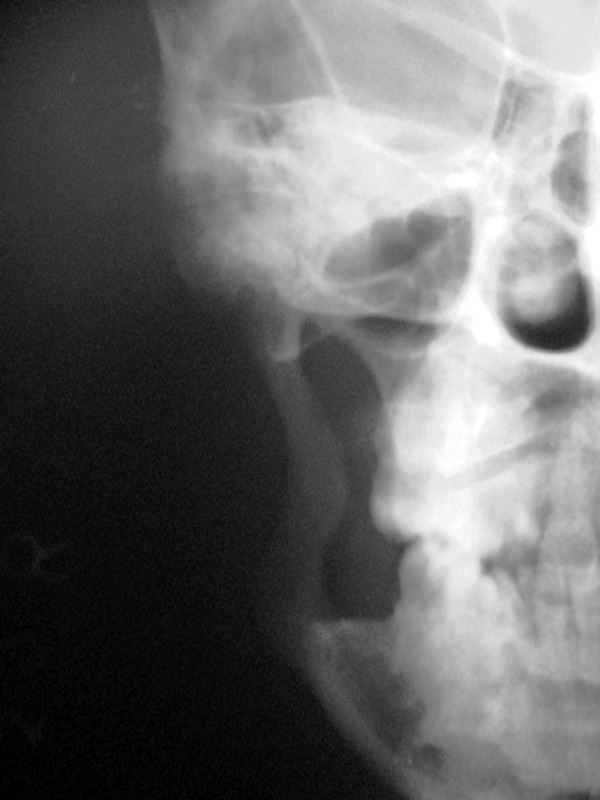
PA view showing stump of posterior border of ramus

CT scan of the recurrent lesion in the right temporal and infratemporal region shows a bony cystic lesion extending from coronoid and condylar region inferiorly to approximately superficial temporal line superiorly. Anteroposteriorly involving complete infratemporal and temporal region. Zygomatic arch is displaced laterally due to expanding bony cyst. Condylar head is fused to posteroinferior portion of the bony cyst (decreasing mouth opening). There is no bony wall inferiorly and is continuous with soft tissue. Content inside the cystic lesion shows radiodensity equivalent to fluid. Cyst lining cannot be described radiologically. Bones forming boundaries of infratemporal region appear normal. No intracranial extension noted radiologically (Figs 4 to 6).

### Aspiration

Preoperatively fluid was aspirated from the bony cystic lesion. Fluid sample was sent for biochemistry for protein analysis, microbiology for culture and cytology for cells. Protein more than 4 mg/dl, culture reported no growth and cytology reported only macrophages.

### Surgical Procedure

**Fig. 7.**
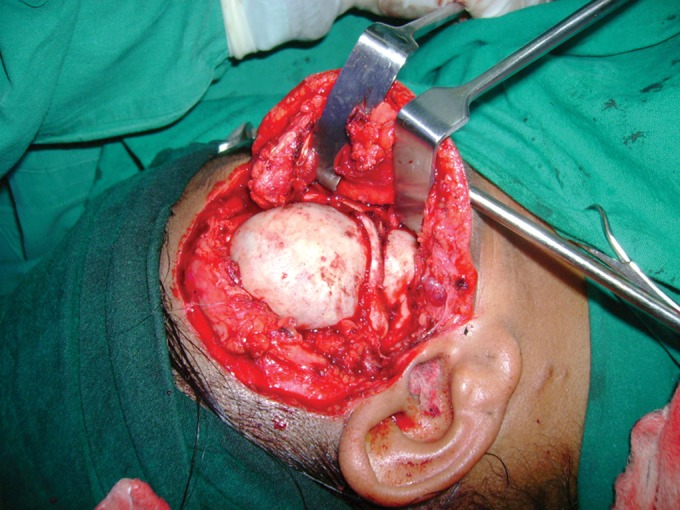
Intraoperative view

**Fig. 8.**
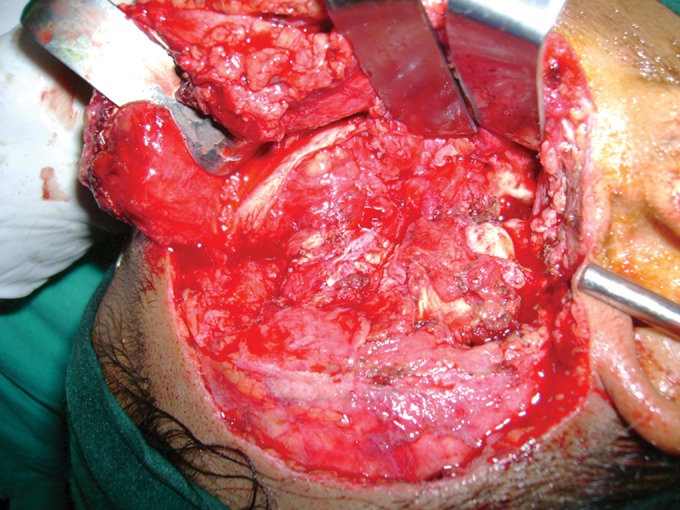
Enmass enucleation of cystic lesion

**Fig. 9.**
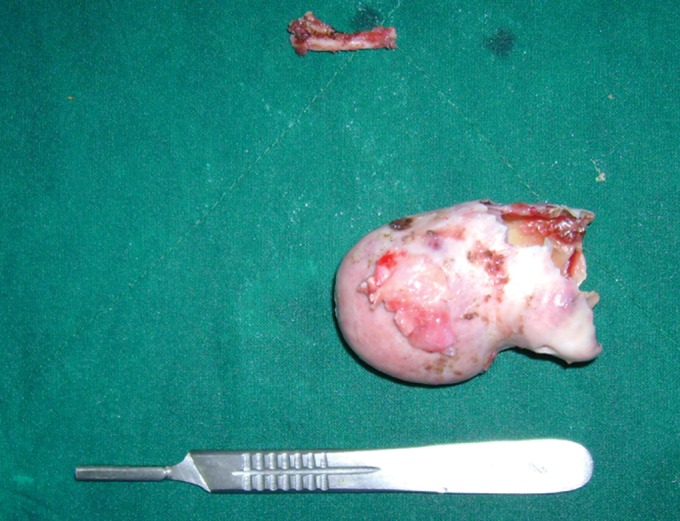
Excise lesion and zygomatic arch

**Fig. 10.**
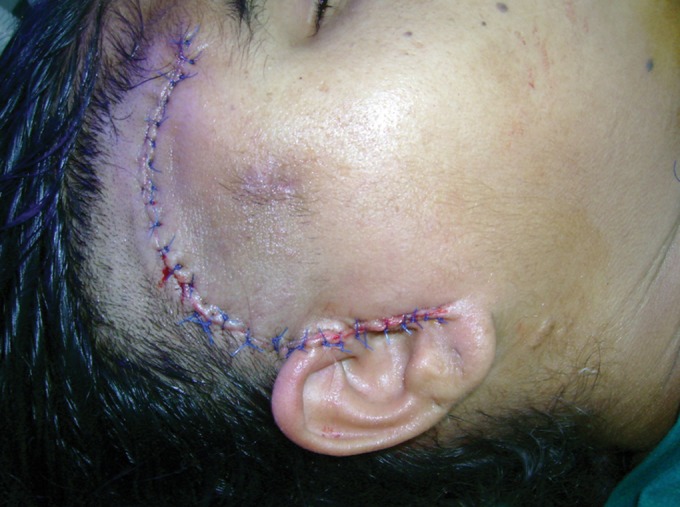
Closure of operative area

**Fig. 11.**
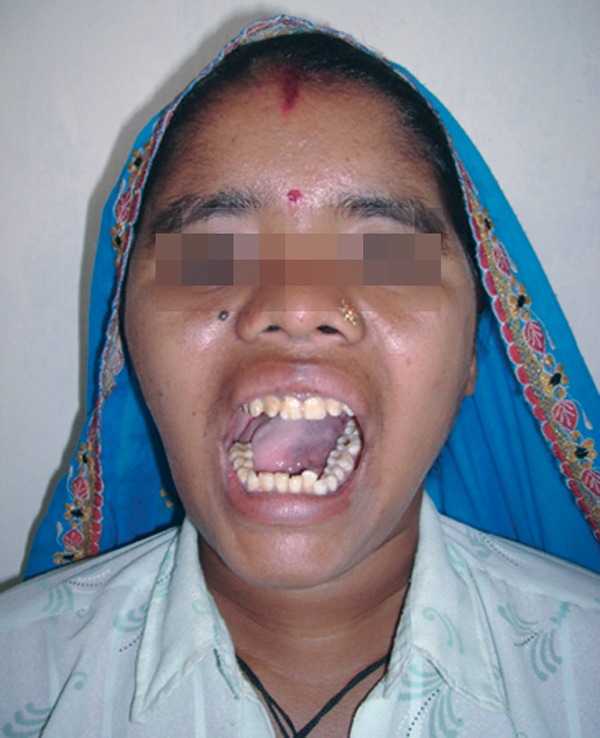
Postoperative improved mouth opening

**Fig. 12.**
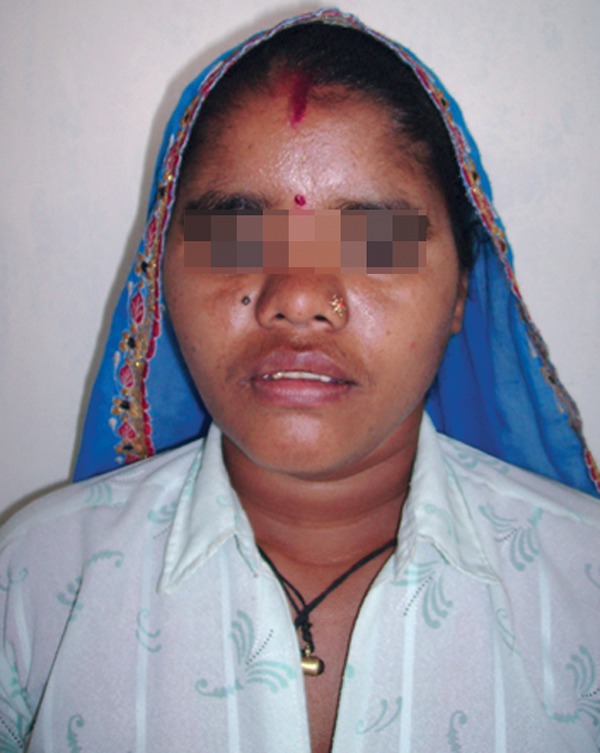
Postoperative view showing complete healing

No preoperative biopsy was planned because clinical, radiological and cyst aspiration findings suggested of benign cystic lesion. Histopathological report of previous surgery was reported as radicular cyst. A preauricular incision extended superiorly in to the scalp around the superior border of cyst up to the lateral part of the forehead anteriorly was placed. Temporal fascia and muscle was identified and incised to expose the bony cyst. Anteriorly pericranium was incised to expose lateral orbital rim. Inferiorly zygomatic arch was identified and dissection carried out inferior to the arch up to the lower border of the bony cyst (Fig. 7). Surprising finding was noted intraoperatively that the bony cyst was located within the temporalis muscle by splitting it in to medial and lateral halves. Part of the muscle was sandwiched between medial portion of the bony cyst and lateral wall of skull. Zygomatic arch was removed. Complete enmass excision of the cyst done after separating it from condylar head (Figs 8 and 9). Condylar head was resected to increase mouth opening. Infratemporal space was carefully examined to find any remnants of cyst lining. Closure was done in four layers (muscle, fascia and pericranium, subcutaneous tissue and skin). Specimen was sent for histopathology and it was reported as unicystic ameloblastoma. Postoperatively patient recovered uneventfully with improved mouth opening (Figs 10 to 12).

**Fig. 13.**
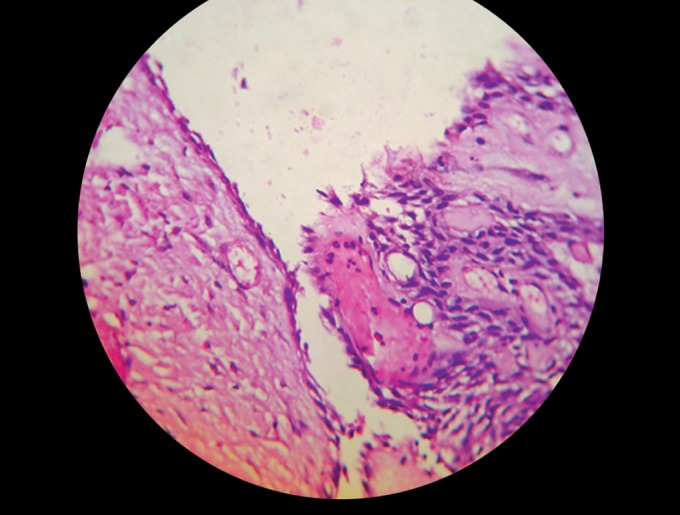
Histological view

### Histopathology (Fig. 13)

The hospital pathologist collected multiple samples from the excised bony cystic lesion. Histological study reported as unicystic ameloblastoma. High-power view shows peripheral columnar cells, resembling preameloblasts, exhibiting reverse polarity.

## DISCUSSION

The ameloblastoma has a long history of recognition, reportage, and controversy. It is the most recorded lesion of the odontogenic series and continues to evoke wide differences of opinion among pathologists and surgeons concerning its position in the neoplastic spectrum, biologic behavior, and management. The ameloblastoma has been described as benign but locally invasive; benign and locally invasive with strong tendency to recur; and locally malignant. The 1992, WHO classification categorizes the ameloblastoma as a benign but locally invasive epithelial odontogenic neoplasm.^1^

Consider the following description of the basal cell carcinoma, an acknowledged malignant tumor: slowly growing, infiltrative, capable of great destruction of soft tissue and bone, recurrent when not completely eradicated, capable of invading vital structures (orbit, skull, brain), seldom metastatic but capable of metastasis, presenting a number of histologic patterns, and arising from the epithelial skin surface and skin adnexa. Substitute oral epithelium for skin surface and dental lamina/enamel organ for skin adnexa, and you are describing the known characteristics of the ameloblastoma.^1^

A unicystic lesion may arise as an ameloblastoma de novo or from secondarily in an odontogenic keratocyst or dentigerous cyst. Occasionally, a unicystic lesion, particularly of the plexiform type, is misdiagnosed as an inflamed cyst with hyperplastic epithelial lining.^1^ A large body of literature has been devoted to explaining the various types of ameloblastomas that arise in association with cysts. Unfortunately, the overall effect of these publications has been to confuse rather than to clarify. Because of the imprecise use of certain terms and their overlapping meanings, the selection of inadequate treatment approaches has sometimes led to unnecessary recurrences.

Ameloblastomas have locally invasive nature so they may spread to the infratemporal fossa, pterygopalatine fossa, parapharyngealspace, orbit, or intracranial space. Ameloblastoma can alsooccur in the temporal region due to implantation of tumor cells in the soft tissues during surgery.^2^ The possible mechanism of spread in this case can be incomplete enucleation of the cystic lining in the coronoid process during previous surgery operated in the regional cancer center.

In this case the lesion did not present with deep seated pain or cranial nerve involvement which is a common presentation of infratemporal masses.^3^ However, this patient only had bony swelling in temporal region and difficulty in opening mouth. However, taking into consideration the severity of lesion, CT scan was performed which revealed a cystic bony lesion involving right temporal and infratemporal region. The inferior portion of the lesion was involving the condylar head of the mandible leading to reduced mouth opening.

Anatomically, the infratemporal fossa lies on the lateral aspect of the cranial base, deep to the zygomatic arch, masseter and mandibular ramus. The infratemporal space or fossa is basically pyramidal in shape. The base of the pyramid is formed by the medial aspect of the ramus and the upper surface of the pyramid is the floor of the skull. The anteromedial aspect corresponds to the posterior aspect of the maxilla and the posteroinferior aspect to the pterygomaxillary fascia.^4^ The infratemporal space lies deep to the fascia and when the tumor masses are small, they may not demonstrate any obvious swelling.

Cohen et al^5^ aptly described the need for advanced imaging in lesions with infratemporal area spread due to their anatomic complexity and surgical inaccessibility. Treatment of ameloblastoma is usually based on clinical manifestations and radiological features. The value of routine radiographs in determining the extent of the lesion may be limited as the three-dimensional spread may not be apparent. Cohen et al^5^ described four cases of mandibular ameloblastoma that were studied using axial and coronal CT. According to the authors, the soft-tissue mass, destruction of cortical bone, and extension of tumor into the infratemporal fossa and adjacent structures were clearly visualized. The features they studied clearly demonstrated the superiority of CT over conventional radiography in delineating the extent of the lesion in all cases.

The surprizing finding of this lesion intraoperatively was splitting of temporalis muscle in to medial and lateral halves by the growing bony cystic lesion. This shows the local infiltrating behavior of the ameloblastoma. The problem of recurrence following removal of an ameloblastoma can be attributed to inadequate excision and the spread of residual tumor fragments within adjacent bone.^6^ In this case previous preoperative incisional and postoperative excisional biopsy from a regional cancer center was reported as radicular cyst. Unicystic lesion, particularly of the plexiform type, is misdiagnosed as an inflamed cyst with hyperplastic epithelial lining. This histological finding would have lead to underestimation of the lesion by surgeon and thus inadequate excision of the lesion. Literature shows that development of an ameloblastoma from an odontogenic cyst takes long period of time. In this case patient presented within 18 months after previous surgery. The possible reason for development of this lesion can be due to the inadequate excision of the cystic lining from the coronoid process of the mandible due to misleading histological presentation of this lesion.
